# The effect of oral ondansetron on QT interval in children with acute gastroenteritis; a retrospective observational study

**DOI:** 10.1186/s12887-021-02937-0

**Published:** 2021-11-10

**Authors:** Heewon Yang, Woochan Jeon, Yura Ko, Sooin Jeong, Jisook Lee

**Affiliations:** 1 Department of Emergency Medicine, Ajou University School of Medicine, 164, World cup-ro, Yeongtong-gu, Suwon, 16499 Republic of Korea; 2 Department of Emergency Medicine, Inje University, Ilsan Paik Hospital, Goyang, Republic of Korea; 3 Division of Pediatric Cardiology, Department of Pediatrics, Ajou University School of Medicine, Suwon, Republic of Korea

**Keywords:** Ondansetron, QT prolongation, Vomiting, Child, Emergency department

## Abstract

**Background:**

In mildly to moderately dehydrated patients with acute gastroenteritis (AGE), oral rehydration therapy (ORT) is the treatment of choice. Though ondansetron is a very effective antiemetics and leads to succeed ORT, there have been reports QT prolongation in patients using it. We investigated the effect of oral ondansetron on QT interval in mildly to moderately dehydrated children with AGE.

**Methods:**

This retrospective observational study was conducted in a single pediatric emergency department (ED) of a tertiary university hospital. We collected the medical records of patients with a primary diagnosis of AGE who received oral ondansetron and underwent an electrocardiogram between January 2017 and June 2018. A pediatric emergency physician calculated the corrected QT interval (QTc) by Bazett’s method, and the calculations were reviewed by a pediatric cardiologist. QTc values before (preQTc) and after (postQTc) ondansetron administration were analyzed. ΔQTc was calculated as the change from preQTc to postQTc. We also investigated any cardiac complications from oral ondansetron.

**Results:**

Total 80 patients were included. The mean age of the patients was 53.31 ± 32.42 months, and 45% were male. The mean dose of oral ondansetron was 0.18 ± 0.04 mg/kg. The mean interval from administration of ondansetron to performance of the electrocardiogram was 65 ± 26 min. The mean preQTc was 403.3 ± 24.0 ms, and the mean postQTc was 407.2 ± 26.7 ms. Two patients had a preQTc ≥460 ms, and one patient had a postQTc ≥460 ms. ΔQTc was ≥30 ms in seven patients (8.8%). No ΔQTc was ≥60 ms. No pre- or postQTc was ≥500 ms. No patient had a fatal cardiac arrhythmia after taking ondansetron.

**Conclusion:**

Oral administration of a single dose of ondansetron in children with AGE did not cause high-risk QTc prolongation or fatal arrhythmia.

## Background

Acute gastroenteritis (AGE) is one of the most common diseases of children, with 1.3 million deaths worldwide annually and about 500,000 deaths of children under 5 years old [1]. The World Health Organization, the US Centers for Disease Control and Prevention, and the American Academy of Pediatrics have recommended oral rehydration therapy (ORT) as initial treatment in mildly to moderately dehydrated children. Successful treatment with ORT reduces the need for intravenous (IV) fluid therapy and can reduce the duration of hospital stay and prevent unnecessary use of medical resources [2, 3]. Vomiting control is pivotal in the treatment of mildly to moderately dehydrated children with AGE. In the emergency department (ED), vomiting is a major obstacle to the use of ORT. Recent studies have recommended the use of oral ondansetron as an antiemetic [4].

Ondansetron is an antagonist of 5-hydroxytryptamine type 3 and may cause QT prolongation due to its mechanism of action. Many studies have reported that ondansetron can enable the successful use of ORT by suppressing vomiting [2, 5–8]. Ondansetron has also been safely and effectively used in patients with cancer and postoperative patients [9]. However, the US Food and Drug Administration reported in 2011 that IV ondansetron possibly caused fatal arrhythmias in patients with prolonged QT interval [10]. On the other hand, recent studies found that IV ondansetron did not cause QT prolongation or increase the risk of fatal arrhythmias in mildly to moderately dehydrated pediatric patients with AGE [9, 11, 12].

For convenience, oral ondansetron has been preferred to IV ondansetron to treat children with vomiting while receiving ORT [13]. Most previous studies have focused on the safety of IV ondansetron, and there have been no studies of changes in QT interval after oral administration of ondansetron. Therefore, we conducted a study to investigate the effect of oral ondansetron on QT interval in mildly to moderately dehydrated children with AGE.

## Methods

### Patients and setting

This retrospective observational study was conducted in a pediatric ED of Ajou University Hospital which is the largest tertiary referral university hospital located on Southern Kyounggi Province. We reviewed the charts of all patients whose primary discharge diagnosis was AGE between January 2017 and June 2018. Among them, we selected patients aged from 6 months to 14 years. Finally, we included patients who received oral ondansetron with ORT and underwent an electrocardiogram (ECG) according to the ORT protocol in our pediatric ED. Patients were excluded if they had clinical dehydration scale (CDS) ≥ 6, oliguria, surgical abdomen, medical history of abdomen surgery, congenital heart disease, arrhythmias, or a history of use of QT-prolonging medication [14]. We also excluded patients with missing medical records, those who could not measure the QT intervals due to ECG noise and those who received intravenous fluid or other antiemetics. The study was approved by institute’ ethics committee. The study also complied with the principles of the Helsinki Declaration.

### ORT protocol

In our pediatric ED, the ORT protocol with oral ondansetron has been performed for mildly to moderately dehydrated patients with CDS ≤ 5 with AGE. If the patient complains of vomiting, 2 mg (for patients 8–15 kg), 4 mg (for patients 15–30 kg), or 8 mg (for patients > 30 kg) of ondansetron (Zofran Zydis®, Novartis, Basel, Switzerland) was administered by the oral route for antiemetic effect [2]. ORT was started 15 to 20 min after administration of oral ondansetron. For early detection of QT prolongation and fatal arrhythmias, ECG recording (lead II; standard paper speed of 25 mm/sec, standard calibration of 10 mm/mV) was performed using biphasic defibrillator (Philips Medical Systems, Andover, MA, USA) before the administration of ondansetron. To detect any changes in the ECG after administration of ondansetron, a post-ECG was obtained before discharge from ED.

In this study, a pediatric emergency physician received 6 h of training from a pediatric cardiologist in the interpretation of pediatric ECGs, in particular, of QT interval measurement. The pediatric emergency physician calculated the corrected QT interval (QTc) by Bazzet’s method [15, 16]. All calculated QTcs were reviewed by the pediatric cardiologist. The pediatric emergency physician and the cardiologist were blinded to whether pre or post-ECG to take ondansetron and analysis of all results. Following the recommendation of a pediatric cardiologist, QT measurement was made in lead II and QT interval was determined as a mean value derived from at least 3 to 5 heartbeats [16]. We also measured the QT interval using the tangent method originally described by Lepeschkin and Surawicz [17]. No prominent U waves or inverted U waves were found on the ECGs.

We summarized the demographic characteristics of the patients, the dose of ondansetron according to body weight, the interval before performing post-ECG, and the parameters of QTc intervals. Also, we reviewed any adverse effects staying the ED.

Our primary outcome was the change in QTc interval (ΔQTc) after ondansetron administration. The definition of QT prolongation was 460 ms and QTc ≥500 ms indicated marked QT prolongation [16, 18]. According to previous “thorough QT study” criteria, ΔQTc ≥30 ms is defined as a “possible” cause for concern and ΔQTc ≥60 ms as a “definite” cause for concern [12, 19, 20]. The secondary outcome was adverse electrical cardiac events after drug administration.

### Data analysis

Continuous variables following normal distribution were expressed as means ± standard deviation. Variables that were not found to follow a normal distribution were expressed as medians and interquartile ranges. Categorical variables were expressed as frequencies and percentages. A comparison of QTc before and after ondansetron administration was analyzed by Student’s paired *t*-test and Mann-Whitney U test on the resulting sample values. SPSS version 15.0 (SPSS, Chicago, IL, USA) was used for all statistical analyses. A *p* value < 0.05 was considered to indicate a significant difference.

## Results

A total of 80 children were included in the analysis (Table 1). The mean age of the children was 53.3 ± 32.4 months (range, 7 to 161 months), and 45% were male. The mean weight was 18.9 ± 10.0 kg. The mean number of vomiting was 2.5 ± 2.2. No patients complained bloody or bilious vomiting. Six patients reported one or two events of diarrhea. The average of CDS was 2 score. The mean dose of ondansetron was 0.18 ± 0.04 mg/kg. The mean interval from oral administration of ondansetron to performance of the ECG was 65 ± 26 min. The mean length of stay in the ED was 147 ± 45 min. According to ED protocol, all of 80 patients received only single dose of ondansetron with successful ORT. No patient was treated by IV hydration or got blood tests other than ORT. The median QTc at baseline (preQTc) and after taking ondansetron (postQTc) was 403.3 ± 24.0 and 407.2 ± 26.7 ms, respectively; the difference was not significant (*p* = 0.08) (Table 2).

**Table 1:** Baseline characteristics (80 patients)

	Mean ± SD	Range
Male	36 (45%)	
Age, months	53.3 ± 32.4	7 – 161
Weight, kg	18.9 ± 10.0	8.0 – 67.9
Number of vomiting	2.5 ± 2.2	1 – 10
Clinical dehydration scale	2.0 ± 1.1	2 – 5
Ondansetron, mg/kg	0.2 ± 0.0	0.1 – 0.2
Time to take post-ECG, min	65 ± 26	30 – 192
Length of stay in ED, min	147 ± 45	143 – 338

Data are presented as number (n, %) or mean ± standard deviation (SD) with range

*ED* emergency department

**Table 2:** The effect of oral ondansetron on QT interval

	Mean ± SD	Range	
PreQTc, ms	403.3 ± 24.0	343.0 – 482.0	
PostQTc, ms	407.2 ± 26.7	343.0 – 472.0	
**Paired t-test**			**95% CI (*P*** **value)**
ΔQTc, ms	3.9 ± 19.9	−40.0 – 47.0	−0.5 – 8.3 (0.08)

Data are presented as mean with standard deviation (SD), Range and Confidential interval (CI). QTc corrected QT interval

QTc is calculated by Bazett’s method

ΔQTc is the change of QT interval before and after oral ondansetron administration

**Fig. 1:**
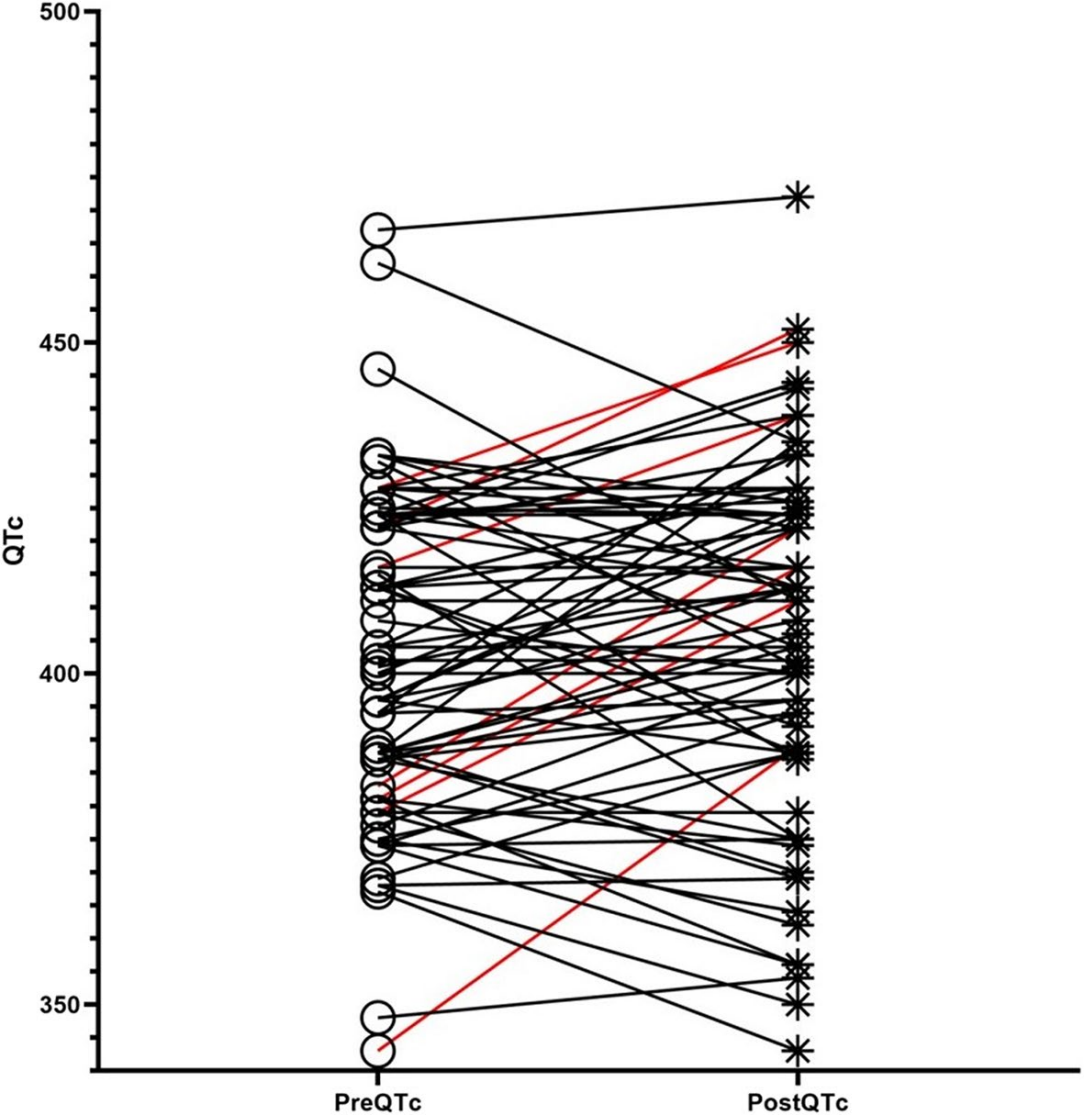
Change of QT interval before and after oral ondansetron administration. Pre and Post QTc value before and after ondansetron administration, respectively; ΔQTc ≥30 ms (red); ΔQTc is the change of QT interval before and after oral ondansetron administration

**Fig. 2:**
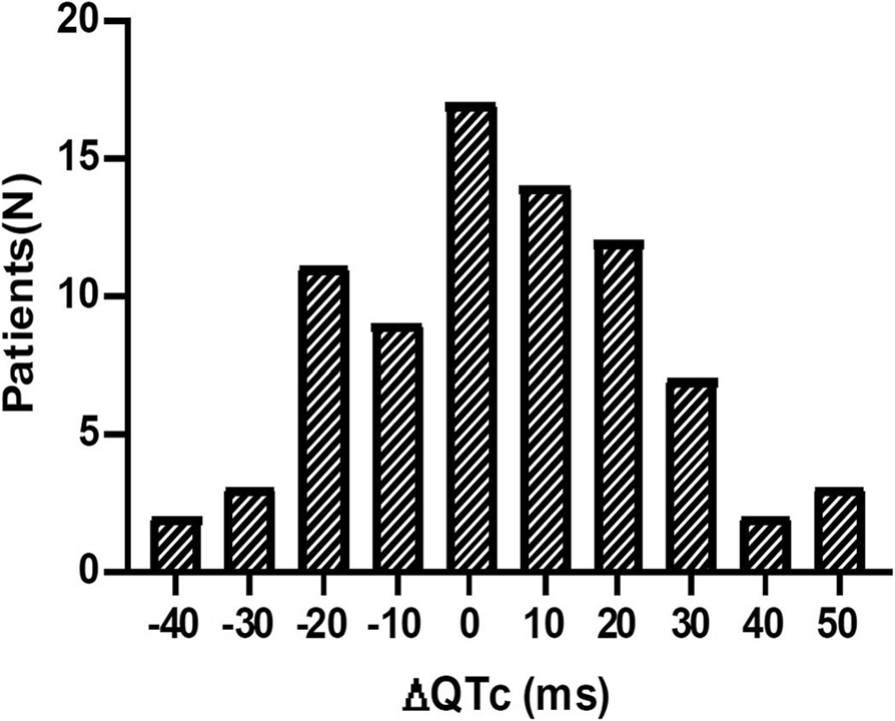
The distribution of ΔQTc by pre and post ondansetron effect

We analyzed the changes in QT interval before and after administration of oral ondansetron (Fig. 1). No patient had a prolonged QTc ≥500 ms. Only two patients had baseline QTc exceeding 460 ms. All of them had no underlying disease and did not take QT prolonging medications. Patient 1 was a 17-month-old girl. She weighed 11 kg and received 2 mg of oral ondansetron. An ECG was tested within an hour and 8 minutes of the ondansetron administration. Her QTc increased from 467 ms to 472 ms with normal sinus rhythm. Length of stay in ED was 3 h 05 min. Patient 2 was a 40-month-old girl. She weighed 15.8 kg and received 4 mg of oral ondansetron. After 58 min we obtained ECG of the patient 2. QTc was decreased from 462 ms to 435 ms with a normal sinus rhythm. Length of stay in ED was 2 h 15 min. After ORT, there was no abnormal rhythm or vital signs. They improved initial symptoms and were safely discharged. A comparison of QTc before and after ondansetron administration (ΔQTc) was performed (Fig. 2). The mean ΔQTc was 3.9 ms (range, − 40.0 to 47.0 ms). Seventy-three children (91.3%) had a ΔQTc < 30 ms, and 7 children (8.7%) had a ΔQTc ≥30 ms. No child had a prolonged ΔQTc ≥60 ms. To analyze the relationship between ΔQTc and the dose of oral ondansetron according to weight, we divided the patients into two groups according to whether ΔQTc was greater or less than 30 ms (Table 3). Patients with ΔQTc ≥30 ms were younger than patients with ΔQTc < 30 ms (median [IQR], 30.0 [10.0 – 44.0] months vs 52.0 [31.5 – 68.3] months, *P* = 0.02). Although post-ECG ΔQTc did not differ between the two groups, ΔQTc of baseline ECG was shorter in the group with ΔQTc ≥30 ms (median [IQR], 383.0 [379.0 – 394.0] ms vs 404.0 [387.8 – 424.0] ms, *p* = 0.04). Seven patients show QT prolongation more than 30 ms. All of them were previous healthy children. Among them, 5 patients (71%) were female. The median dose of ondansetron per weight (kilogram) and the interval between administration of ondansetron and performing the post-ECG did not significantly differ between the two groups. During the stay in the ED, there were no adverse cardiac events or fatal arrhythmias after taking the medicine.

**Table 3:** Comparisons according to ΔQTc

	ΔQTc < 30 ms	ΔQTc ≥30 ms	*P*
Patients, n (%)	73 (91.3)	7 (8.7)	
Female, n (%)	39 (53)	5 (71)	
Age, mon	52.0 (31.5 – 68.3)	30.0 (10.0 – 44.0)	0.02
Weight, kg	17.0 (13.0 – 20.0)	12.0 (10.6 – 15.6)	0.02
Ondansetron, mg/kg	0.2 (0.1 – 0.2)	0.2 (0.1 – 0.2)	0.56
PreQTc, ms	404.0 (387.8 – 424.0)	383.0 (379.0 – 394.0)	0.04
PostQTc, ms	411.0 (388.0 – 425.3)	422.0 (411.0 – 439.0)	0.09

Data are presented as number (n, %) or median with interquartile range (IQR)

ΔQTc is the change of QT interval before and after oral ondansetron administration

*P* value < 0.05 was set to be statistically significant

## Discussion

To the best of our knowledge, this study was the first to evaluate the effect of single dose oral ondansetron on changes in the QT interval. We showed that single dose oral ondansetron did not prolong the QT interval and resulted in effective use of ORT in children with AGE. A previous study reported that IV ondansetron did not affect the QTc of pediatric patients who were receiving ondansetron for nausea and vomiting [9]. According to the author’s description, oral administration of ondansetron was crucial for ORT in mild to moderately dehydrated children. Though this study was retrospectively small sample sized, our results were noteworthy in this respect.

The patients in this study had a mean age of 53.3 ± 32.4 months, ranging from 7 to 161 months. This was an appropriate age group to receive ORT with oral ondansetron. The size of the single dose of ondansetron varied according to the patient’s weight: 2 mg for patients 8 – 15 kg, 4 mg for patients 15 – 30 kg, and 8 mg for patients > 30 kg. The mean dose per unit weight was 0.18 ± 0.04 mg. Because the bioavailability of the oral form of ondansetron is 56 to 60% of that of IV ondansetron due to first-pass metabolism, the size of the dose was adequate, considering the recommended IV dose of 0.1 to 0.15 mg/kg [9, 12]. The mean interval from oral administration of ondansetron to performance of the EGG was 65 ± 26 min. Since the time to reach peak blood concentration of ondansetron is 30 to 120 min after oral ingestion, our results showed the change in QTc at peak concentration of oral ondansetron [21].

A previous study of IV ondansetron in healthy children who were undergoing elective surgery under general anesthesia or who had AGE with mild dehydration found a nonsignificant prolongation of QTc of 0.4 to 17 ms [22]. Our results were similar, showing that oral ondansetron did not prolong QTc and that changes in ΔQTc were not significant. However, Trivedi et al. reported that several risk factors, including electrolyte imbalance, multidrug use with the possible risk of QT interval change, and organ dysfunction, prolonged QT in pediatric patients in the intensive care unit [18]. In patients who had risk factors or baseline QTc over 460 ms, QT could be prolonged more than 500 ms. The patients we included were children with acute diseases who were previously healthy and without underlying disease and who received only a single dose of ondansetron. Recently two pediatric emergency centers in Pakistan conducted randomized, double-blind, placebo-controlled trial that supported our results which reported safety use and improve success rate of ORT of single dose oral ondansetron among children with gastroenteritis-associate vomiting [23]. Therefore, further multicenter prospective study focusing on the effect of oral ondansetron on QT prolongation is needed to deduce the efficacy and safety of repeated doses of ondansetron in patients with a high risk of QT prolongation.

The International Conference on Harmonization (ICH) findings have guided the US Food and Drug Administration on issues of drug-induced QT prolongation. The ICH has suggested that an increase in QTc of 30 ms is a “possible” cause for concern and an increase of 60 ms is a “definite” cause for concern. After administration of an at-risk drug, increases in QT or QTc to ≥500 ms or to ≥60 ms over baseline are commonly used as thresholds for potential discontinuation or alternative pharmacotherapy [16, 18]. In our study, there were seven patients with an increase in QTc of ≥30 ms and no patients with an increase of ≥60 ms. Patients with an increase of ≥30 ms were younger and had a lower body weight. In patients with an increase of ≥30 ms, baseline QTc was shorter and the change of QTc after taking medicine was greater. The dose of ondansetron per unit weight and the interval from oral administration of ondansetron to performance of the EGG did not significantly differ between patients who had an increase of < 30 ms and those who had an increase of ≥30 ms.. However, ondansetron might cause a greater prolongation of QTc in younger children. Although all patients had QTc < 460 ms and there was no “definite” concern about ΔQTc, physicians need to be careful when using ondansetron in younger children.

Our study had several limitations. First, because it was a retrospective analysis, we could not analyze changes in QTc over time after oral ingestion of ondansetron. A previous study of IV ondansetron showed that the temporarily prolonged QTc became shorter over time after administration [9, 10]. Because the blood concentration of oral ondansetron peaks at 30 to 120 min, further studies should measure QTc over this time range after oral administration. Second, the study had a small sample size. We retrospectively enrolled all patients who were satisfied with the inclusion criteria during the study period. We calculated statistical power analysis to determine sample size. According to the effect of intravenous ondansetron on QTc study among the children with AGE, incidence of ΔQTc > 30 ms was 7.5% after IV ondansetron administration [10]. Minimum number for adequate study power sample size was 8 (80% power, α = 0.05). We finally collected and analyzed 80 patient’s data. Although this was retrospective study, our results with 80 participants could have power. However, being conducted in a single ED on acutely ill patients who were previously healthy, the results may not apply to the entire pediatric populations. In this pilot study, only one emergency physician measured QTc and one cardiology specialist confirmed. To improve data quality, we believe that two more physicians need to perform these measurements and check variability and reliability between observers in the further study. Third, there were no follow-up investigations such as ED revisit or outpatient visit. As a result, we were unaware of the long-term side effects. However, given the blood peak time of an oral ondansetron, we thought that the length of stay of ED was sufficient to observe side effects after oral administration. These limitations indicate that a larger prospective study is needed to explore the possibility of QT prolongation by oral ondansetron.

## Conclusion

The effect of oral ondansetron on QT interval is one of the crucial subjects for its safety use among previously healthy children. Our study found that oral administration of a single dose of ondansetron did not result in a significant prolongation of QTc or fatal arrhythmias in mild to moderation dehydrated children with vomiting.

## Data Availability

The study data is available from the corresponding author on reasonable request.

## Abbreviations

AGE: Acute gastroengeritis
ORT: Oral rehydration therapy
CDS: Clinical dehydration scale
IV: Intravenous
ED: Emergency department
QTc: Corrected QT interval
IQR: Interquatile range
